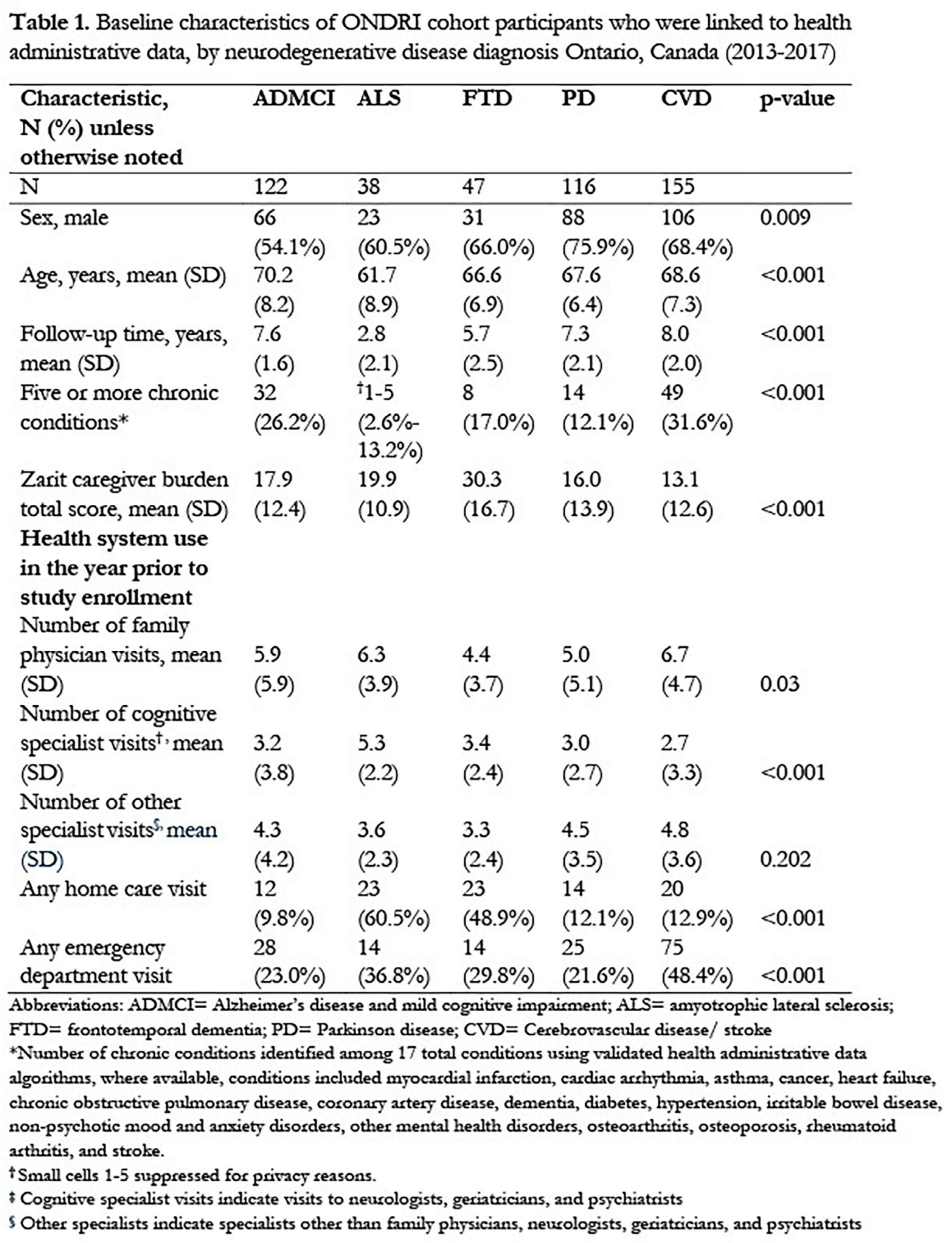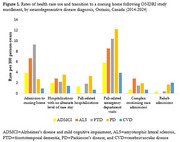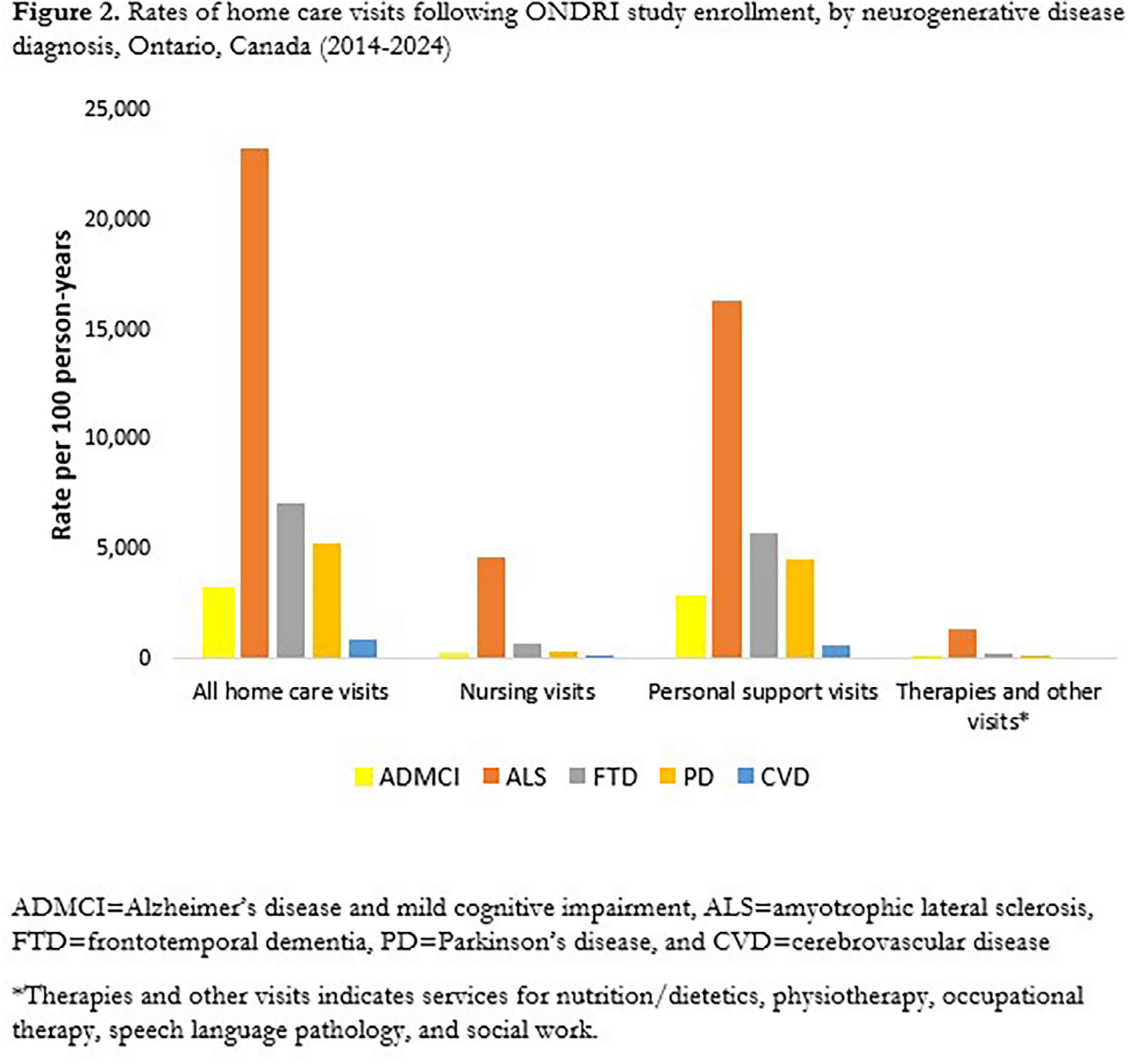# Health service utilization and transition to nursing home among a cohort with mixed neurodegenerative pathologies: the linked Ontario Neurodegenerative Disease Research Initiative (ONDRI) Cohort

**DOI:** 10.1002/alz70860_101213

**Published:** 2025-12-23

**Authors:** Susan E Bronskill, Laura C Maclagan, Lavina Matai, Abby L Emdin, Angela C. Roberts, Malcolm Binns, Paula M McLaughlin, Sandra E. Black, Richard H. Swartz

**Affiliations:** ^1^ ICES, Toronto, ON, Canada; ^2^ Sunnybrook Research Institute, Toronto, ON, Canada; ^3^ Dalla Lana School of Public Health, University of Toronto, Toronto, ON, Canada; ^4^ Western University, London, ON, Canada; ^5^ Rotman Research Institute, Baycrest Academy for Research and Education, Toronto, ON, Canada; ^6^ Nova Scotia Health, Halifax, NS, Canada; ^7^ Dr. Sandra E. Black Centre for Brain Resilience and Recovery, LC Campbell Cognitive Neurology, Hurvitz Brain Sciences Program, Sunnybrook Research Institute, University of Toronto, Toronto, ON, Canada; ^8^ University of Toronto, Toronto, ON, Canada; ^9^ Sunnybrook Health Sciences Centre, University of Toronto, Toronto, ON, Canada; ^10^ Sunnybrook Research Institute, University of Toronto, Toronto, ON, Canada; ^11^ Sunnybrook Health Sciences Centre, Toronto, ON, Canada

## Abstract

**Background:**

The Ontario Neurodegenerative Disease Research Initiative (ONDRI) was a prospective cohort study that enrolled persons with varied neurodegenerative pathologies including Alzheimer's disease and/or mild cognitive impairment (ADMCI), Parkinson's disease (PD), amyotrophic lateral sclerosis (ALS), frontotemporal dementia (FTD), and cerebrovascular disease± cognitive impairment (CVD) who underwent clinical and functional assessments in Ontario, Canada. We sought to examine long‐term differences in health system use, transition to nursing home, and mortality across neurodegenerative pathologies via linkage with provincial health administrative data.

**Methods:**

Of 520 participants enrolled in the ONDRI study, 478 provided consent and were linked to health administrative databases. This included 122 persons with ADMCI, 38 with ALS, 47 with FTD, 116 with PD, and 155 with CVD. Participants were followed from the year prior to their enrollment date (2014‐2017) to March 31^st^ 2024, death, or loss of health insurance eligibility, whichever occurred first. Rates of health service use, transition to nursing home, and mortality were calculated by neurodegenerative pathology and expressed per 100 person‐years.

**Results:**

Persons with PD were most likely to be male, while those with ADMCI showed the lowest proportion males (75.9% vs. 54.1%). Those with ADMCI were older compared to persons with ALS (mean 70.2 vs. 61.7 years). Follow‐up time ranged from an average of 2.8 years in persons with ALS to 8.0 years in those with CVD. Persons with ALS had the highest mortality rate, while those CVD had the lowest (35.3 vs. 2.4 per 100 person‐years). Persons with all neurodegenerative diseases showed a high use of health services, varying by service type. Persons with ALS showed the highest rate of home care visits, including visits for nursing, personal support, and therapies, followed by persons with FTD. Persons with PD had the highest rate of fall‐related emergency department visits and hospitalizations. Persons with FTD had the highest rate of transition to nursing home.

**Conclusions:**

Our findings demonstrate that persons with neurodegenerative pathologies require different health services, with varying intensity, to meet their needs. Linkage of health administrative data to clinical cohorts supports examination of health service milestones and allows for long‐term follow‐up at relatively low cost.